# Impact of Silicon-Based Biostimulant on Improving Growth and Morpho-Physiological Traits of Sweet Basil (*Ocimum basilicum* L.) in a Glasshouse Production System

**DOI:** 10.3390/plants15060859

**Published:** 2026-03-10

**Authors:** Zoia Arshad Awan, Michael T. Gaffney, Lael Walsh

**Affiliations:** Horticulture Development Department, Teagasc Food Research Centre, Ashtown, D15 KN3K Dublin, Ireland; michael.gaffney@teagasc.ie

**Keywords:** basil (*Ocimum basilicum* L.), biostimulants, silicon, controlled environments, horticulture

## Abstract

Silicon-based biostimulants are gaining increasing interest for their ability to enhance plant performance and stress tolerance. In protected cultivation, where environmental conditions are already carefully managed, it remains unclear whether adding biostimulants provides meaningful benefits and how they should be used. This study examines whether silicon (Si) biostimulants can enhance the growth and morpho-physiological traits of sweet basil (*Ocimum basilicum* L.) in glasshouse production and which application rates are most effective. Two Si-based products with similar silicon content and different formulations were applied as soil drenches at four rates (10 mL, 100 mL, 1 L, and 2 L per hectare). Plant growth, biomass, photosynthetic performance, and physiological traits including membrane stability and electrolyte leakage were measured. Overall, silicon treatments improved most traits compared with untreated plants. Basil receiving Si showed longer shoots and roots, greater fresh and dry weight, and healthier leaves with better photosynthetic activity, as reflected by higher SPAD values and chlorophyll content. The response often depended on the dose: lower rates (10 mL and 100 mL h^−1^) of the silicic acid tetraethyl ester (21% Si) led to clear improvements in 7 of 12 measured traits, while higher rates (especially 2 L ha^−1^) reduced leaf size and morphology. However, root length: shoot length ratios were low across all treatments with the second biostimulant product: SiO_2_ with chelated iron (T5–T9). Certain results are paradoxical, suggesting a trade-off in growth and defense. In some instances, low doses promote growth but potentially worsen some physiological indicators, while high doses inhibit growth but improve stress resistance indicators. The conclusion indicates that silicon-based biostimulants are valuable to include in single-harvest basil production systems, when applied at a suitable rate. Choosing the correct formulation and dose requires testing and optimization to the crop and growing system.

## 1. Introduction

Sweet basil (*Ocimum basilicum* L.) is a member of the Lamiaceae family and is widely cultivated worldwide as an aromatic herb for culinary and therapeutic uses [1,2]. Its high content of natural antioxidants and phytopharmaceuticals has led to its traditional use in folk remedies for several ailments [3,4], and it is widely regarded as a functional food. Cultivation can be challenged by biotic and abiotic stresses, which often limit the productivity and quality of basil crops in the field [5,6]. Hence, the cultivation of basil is increasingly shifting toward controlled-environment and hydroponic systems to meet demand for high-quality produce [7]. Various studies aim to enhance basil cultivation by developing strategies to mitigate environmental stresses, including drought, salinity, temperature fluctuations, and diseases [8,9]. Recent studies have explored the potential of silicon supplementation as an effective strategy to enhance crop production and improve crop resilience under biotic and abiotic stress conditions [10,11,12,13,14].

Silicon (Si) is classified as a beneficial element for plants, playing a crucial role in regulating physiological and biochemical processes that enhance crop growth and development [15]. Si significantly enhances photosynthesis, maintains ion homeostasis, activates the antioxidant defense system, regulates genes involved in various physiological processes, and promotes the synthesis of secondary metabolites [16]. Other published studies have shown that Si improves plant resilience to drought, salinity, and nutritional deficiencies by enhancing water-use efficiency, osmotic control, and antioxidant activities [17,18]. Si also modulates phytohormone synthesis, which is crucial for regulating growth and stress responses in plants [19]. The deposition of Si in plant tissues strengthens cell walls, providing mechanical support and defense against biotic stressors such as pests and pathogens [20]. Additionally, enhancing plant stress tolerance through silicon supplementation is linked to increased antioxidant capacity [2,21] and modifications in isozyme patterns [22]. The beneficial effects of silicon foliar application have been well-documented, including its role in enhancing plant growth and increasing biomass production [23]. A previous study revealed that Si not only enhances plant growth and yield but also improves quality traits, such as aroma and phytochemical content, in leafy vegetables and herbs, including basil [24]. This indicates a promising role of Si in improving both agronomic performance and morpho-physiological attributes in basil cultivation.

Alternative studies also indicate that the effects of Si supplementation on plant growth and stress tolerance can vary significantly with the rate of application. Optimal Si concentrations can enhance plant resilience and reduce oxidative stress by augmenting activities of antioxidant enzymes (e.g., superoxide dismutase and catalase) and increasing the accumulation of osmolytes, such as proline and soluble sugars, under drought or salinity stress [25,26]. Application rates vary with plant species and cultivation methods. Foliar applications typically range from 50 to 100 ppm, while soil applications can vary from 12 kg/ha to 300 kg/ha, depending on the crop [12]. Notably, inappropriate levels of Si supplementation may yield negligible benefits at low application rates or toxic effects at high rates, thereby impeding plant growth and disrupting metabolic activity [27]. The adverse effects are often associated with overaccumulation of Si in plant tissues, which can cause nutrient imbalances and disrupt normal physiological processes [28]. Such findings underscore the importance of applying Si at appropriate application rates to maximize its benefits while minimizing potential adverse effects.

The role of Si-based biostimulants in crop cultivation has been extensively studied in rice and wheat [25,28] and, to a lesser extent, in leafy greens. However, there are indications that Si application may be beneficial for spinach [29] and basil production in high-salt environments [30,31]. Studies integrating Si-based biostimulants into standard crop production cycles, outside of biotic and abiotic stress evaluation, remain scarce. Yet interest from regulators, farmers and investors remains high as they seek cost-effective solutions to bolster crop production [32]. Furthermore, complexity in biostimulant modes of action has instead led to research focused on understanding and optimizing “efficacy” and “safety” in support of adoption and integration [33]. Due to the issue that plants grown in glasshouse systems still experience temperature fluctuations due to the external environment [34], e.g., leaf tissue temperature change affects growth and metabolic activity [35], herein lies the value and importance of this research. The sensitivity of basil to temperature change makes it an important experimental crop [36]. Testing Si-based biostimulants as part of standard production protocols with intention to establish efficacy, optimize dosages and avoid toxicity is highlighted in the literature as an area for further research [32].

This research study forms part of “The Leaf No Waste project” funded by Science Foundation Ireland (20/FIP/FD/8934), exploring options to reduce food waste by adopting interventions at the crop production phase [37,38]. With a focus on fresh horticultural crops, including leafy vegetables and herbs, the project assesses the extension of shelf-life following treatment with silicon, which is expected to reduce crop respiration after harvest, without compromising shelf life or contributing to greater levels of food waste [29,37]. As such, plant science and postharvest metrics are evaluated as part of the research study.

In summary, the main objective of this research study is to investigate the potential effects of Si supplementation on the growth and morpho-physiological traits of basil crops produced under controlled glasshouse conditions and further establish if the use of Si influences metrics with a bearing on postharvest quality. Additionally, we seek to determine the optimal Si formulation and supplementation rate for efficient basil production.

## 2. Methods

### 2.1. Experiment Site and Plant Material

A growth trial was designed to cultivate sweet basil in a peat growth medium grown in a glasshouse compartment (temperature: 20–25 °C; relative humidity: 50–70%) at Teagasc, Food Research Centre, Ashtown, Dublin, Ireland (53.379775° N, 6.336400° W). The high-yielding, vigorous commercial basil variety named ‘Sweet Genovese’ [36] which is widely cultivated in Ireland was selected for this experiment. Basil requires sunshine and long-day length for growth, blooming well in summer. Under cultivation, successive harvests are possible in the crop cycle prior to flowering. Growth periods range from 4 to 6 weeks depending on sunshine and light levels.

About fifteen-day-old basil seedlings, which were germinated in a peat-based substrate plug (FlexiPlugs^®^ by PROTEC^TM^ horticultural products, Redland Bay, Australia), were obtained from a local plant supplier using seeds procured from “CN Seeds” (Pymoor, Ely, Cambridgeshire, United Kingdom). Each pot was filled with a peat growth medium (ICL Professional Horticulture: Seed and Modular) composed of sphagnum, moss and peat with a pH of 4.5–4.9 and EC of 269–379 µS/cm, and supplemented fertilizers (N144 P73 K239 per 75 L bag). At the beginning of the experiment, each plug containing 10 basil seedlings (fifteen days old) was transplanted into a filled pot with a volume of 850 mL (0.011 m^2^).

### 2.2. Fertilizers and Si Supplementation

The pot trial experiment took place from 18 June to 12 July 2024. This research utilized two different Si-based biostimulants (Si-bio), commercial products based on pre-trial research (unpublished). Two Si-bio products, (1) Si-bioA: SiO_2_, chelated Fe (20% Silicon) and (2) Si-bioB: silicic acid tetraethyl ester (21% silicon), were applied at 4 different application rates such as 0.01X, 0.1X, 1X and 2X (Table 1): (i) 100 times lower than commercial rate; (ii) 10 times lower than commercial rate; (iii) commercial rate (application rate for leafy greens as per product label instructions) and (iv) double the commercial rate (Table 1). Si supplementation was applied to the crop by drenching the growing media with the required volume for the potted media (50 mL per pot) at the base of the plant, avoiding contact with the basil leaves. All treatments and controls also received liquid fertilizers named Terra Aquatica TriPart^®^ (Biopole, Fleurance, France), named grow, micro, and bloom providing essential macronutrients (nitrogen, potassium, phosphorus) and essential minerals; prepared as a nutrient solution (EC: 1.8) in a ratio of 10:8:4, respectively, as recommended on the product label. The pots received a total of 5 applications of each Si biostimulant (Si-bioA and Si-bioB) (every 3rd day) as well as bi-weekly liquid fertilizer feeds, applied by drenching the growing media.

### 2.3. Experiment Design and Layout

**Figure 1:**
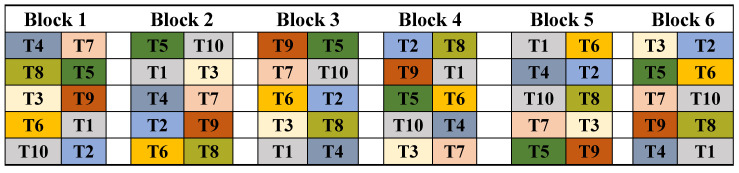
Layout of the randomized block design basil pot experiment located in the glasshouse compartment. Treatments displayed T1: Control (Si non-treated); T2: Si-bioA 0.01X; T3: Si-bioA 0.1X; T4: Si-bioA 1X; T5: Si-bioA 2X; T6: Si-bioB 0.01X; T7: Si-bioB 0.1X; T8: Si-bioB 1X; T9: Si-bioB 2X; T10: Control (Si non-treated), while Si-bioA: SiO2, chelated Fe and S-bioB: silicic acid tetraethyl ester.

Pots were arranged in a completely randomized block design on a raised bench within a glasshouse compartment, with 10 pots per block and 6 blocks in the experiment. A single replicate pot for each of the 8 treatments (2 products × 4 treatments, corresponding to T2–T9) and 2 replicates for the untreated control (corresponding to T1 and T10) were included in each block (Figure 1). Additional replicates of the untreated control (T10) were included to ensure a balanced number of pots and an equitable spacing of pots within each block. Each treatment (including the two controls) was analyzed separately, and additional control treatment was used to assess the effect of pot location.

### 2.4. Studied Attributes

Harvesting commenced when at least 6 true leaves had fully emerged and when plant height had reached ~20 inches. Shoot and root length (cm), root-to-shoot length ratio (cm), number of basil leaves (*n*), shoot fresh and dry weight (g) were recorded as agronomic parameters; relative water content (%), membrane stability index (%) and electrolyte leakage (%) were assessed as physiological parameters, and SPAD index (µg cm^−1^), total chlorophyll content and carotenoids (mg/g of fresh weight) were evaluated as photosynthetic parameters.

#### 2.4.1. Agronomic Parameters

Shoot and root length (cm)

Shoot length was measured at the harvest stage by measuring the tallest plant in each replicate pot (*n* = 6) from the substrate surface to the tallest shoot tip by placing a ruler at the base of the medium at a perpendicular angle.

Roots were also carefully extracted from the peat medium, and the length of the longest root emerging from the peat plug to the end of the root tip was measured in each pot (*n* = 6) by using a ruler.

In addition, the root-to-shoot length ration was calculated for morphological assessment by dividing root length by shoot length.

Number of basil leaves

The number of leaves was counted at the harvest stage and recorded as the total number of leaves for each pot (*n* = 6).

Shoot fresh and dry weight (g)

Basil plants were harvested by cutting the stems 1 cm above the peat substrate. Harvested basil plants from each pot, ±10 plants per pot combined, were placed in an individual brown craft bag. Basil shoot fresh weight (g) was recorded using a weighing balance (as a measure of crop yield) for each bag (*n* = 6).

After measuring the fresh weight of basil shoots, brown craft bags containing the basil plants were placed in a drying oven at 70 °C for 72 h, after which the shoot dry weight (g) was measured for each replicate per treatment.

#### 2.4.2. Physiological Parameters

Relative water content

Before harvest, a single fully expanded basil leaf was randomly collected from one plant per pot, making a random selection from three replicate pots per treatment (*n* = 3 replicate pots). This leaf was weighed and recorded using a delicate balance for fresh leaf weight (FLW), after which it was immediately placed in a 50 mL sealed falcon tube and soaked in distilled water for 24 h at 4 °C. After one day of incubation, the leaf was taken out from the water to record the turgid leaf weight (TLW). The leaf was then placed in filter paper and put in the oven at 70 °C for 24 h to record the dry leaf weight (DLW). Relative water content was calculated using the following equation, as described by Torres et al. [39].

Relative water content %=[(FLW−DLW)⁄(TLW−DLW)]×100

Membrane stability index and Electrolyte leakage

A fully expanded basil leaf was randomly collected from one plant per pot. This was done in three replicate pots, with one pot randomly selected for each treatment (*n* = 3). The analysis of membrane stability index (MSI) and electrolyte leakage (EL) was performed using the method described by Godara et al. [40]. Two sets of basil leaf samples were prepared; each basil leaf was halved, and a 0.1 g portion was weighed, cut into small pieces, and placed in a test tube containing 10 mL of distilled water. The second half of the same leaf was used to prepare an identical second set following the same procedure. One set was incubated at 40 °C for 30 min, and the second set at 100 °C for 15 min. After incubation, one set was used to measure electrical conductivity as EC1 (samples incubated at 40 °C) and another set for EC2 (samples incubated at 100 °C) using an EC meter (HI-2003 Edge Conductivity Meter by Hanna). The MSI and EL were calculated using the formula:

Membrane Stability Index %=[1−(EC1/EC2)]×100

Electrolyte Leakage %=ECI/EC2×100

#### 2.4.3. Photosynthetic Parameters

(a) SPAD index

The relative chlorophyll content of a leaf in terms of leaf SPAD index was measured from a most recently fully expanded basil leaf using a SPAD-502^®^ chlorophyll meter (Minolta Camera Co., Ltd., Osaka, Japan). Three SPAD readings were taken per leaf, which were averaged to obtain a single reading per replicate plant. SPAD index from a plant in each pot (*n* = 6 replicate pots per treatment) was used for this assessment.

(b) Total chlorophyll content and carotenoids

The chlorophyll pigments (Chl *a*, Chl *b*) and carotenoids were determined using a method performed by Awan et al. [41]. Basil leaves were randomly sampled from a single plant in 3 replicate pots per treatment, and 0.5 g of basil leaf was ground in 2 mL of 80% ethanol (*v*/*v*) with the help of a pestle and mortar. The homogenous mixture was centrifuged at 4 °C and 10,000 rpm for 15 min. A supernatant of ethanolic leaf extract was used to measure absorbance at 470 nm for carotenoids, and 645 nm and 663 nm for chlorophyll *a* and chlorophyll *b*, respectively, for assessing the photosynthetic pigments of basil using a UV spectrophotometer (BioTek Epoch microplate spectrophotometer). Total chlorophyll content and carotenoids were calculated against a blank (80% ethanol only) using the formula specified by Lichtenthaler and Wellburn [42].

Chlorophyll a (mg/gFW)=[0.0127(OD663)−0.00269(OD645)(V/W)]

Chlorophyll b (mg/gFW)=[0.0229(OD645)−0.00468(OD663)(V/W)]

Total chlorophyll (mg/gFW)=[(20.2×OD645)+(8.02×OD663)(V/(1000×W)]

$$Carotenoids=[(1000A470-3.27\left(chlorophyll\;a\right)-104\left(chlorophyll\;b\right)]/229$$

### 2.5. Statistical Analysis

All data was subjected to analysis of variance (ANOVA) with the means compared by the Least Significant Difference (LSD) using the software Statistix (version 10) for determining significance at *p* < 0.05.

## 3. Results

**Figure 2:**
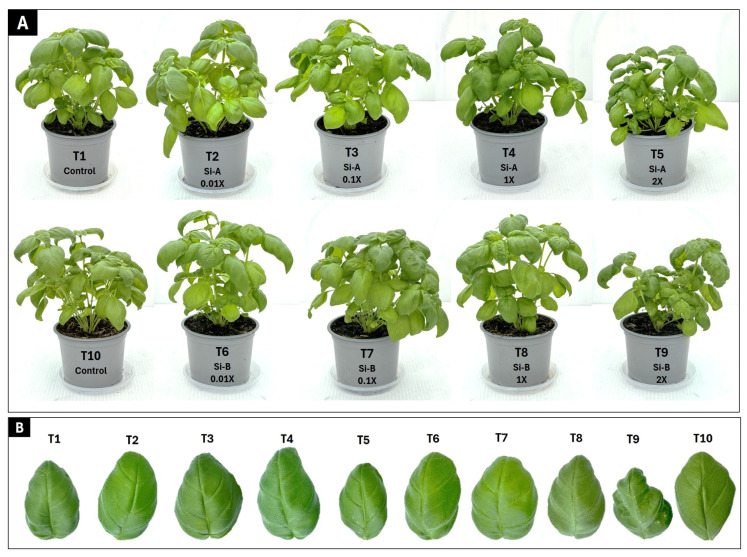
(**A**,**B**) Effect of Si supplementation on the growth and morphology of basil plants growing in peat under glasshouse conditions as affected by two Si-based biostimulants (Si-bio) applied at 4 application rates and non-treatment controls. Treatments: Control: Si non-treated; Si-A (SiO_2_, chelated Fe); Si-B (silicic acid tetraethyl ester).

Visual differences in plant morpho-phenotypic appearance were observed and a significant effect of both biostimulants (bioA and bioB) were noticed in basil growth, specifically in T2, T3, T6, T7 and T8 as compared to controls (T1 and T10) (Figure 2A). Notably, in experiments including preliminary trial (Supplementary File, S1), a greater number of smaller and deformed leaves was observed at a higher application rate (2X) using Si-bioB (Figure 2B).

### 3.1. Agronomic Parameters

#### 3.1.1. Shoot and Root Length

The tallest shoot lengths were recorded at lower Si application rates (T2, T3, T6, and T7) of both products. Compared to controls, shoot lengths with Si-bioB were significantly enhanced by 11%, 9%, 2% and 3% at four application rates, i.e., 0.01X, 0.1X, 1X, and 2X, respectively. For Si-bioA, a similar increasing trend was observed across the 3 application rates, with 9% at 0.01X and 0.1X and 3% at 1X compared with untreated controls. Significantly smaller shoot lengths were recorded in T5 at double the application rate (2X) of Si-bioA. Interestingly, both Si-products (Si-bio) at the commercial (1X) and double commercial application rate (2X) showed non-significant differences in plant shoot length compared to controls (Table 2).

Results for basil root length using Si-bioA at all application rates were greater than controls and also greater than Si-bioB treatments. Significantly increased root lengths were recorded for Si-bioA, by 14%, 17%, and 24% at 0.01X, 0.1X, and 1X, respectively, when compared to untreated controls. In comparison, only the lowest Si-bioB application rate (T6, 0.01X) resulted in a 13% increase in root length relative to controls (Table 2).

Additionally, the root-to-shoot length ratio across all treatments ranged from 0.64 to 0.94 (i.e., <1), indicating that shoot length exceeded root length in every case. The lowest ratios were observed in T7 and T8, reflecting enhanced shoot elongation and canopy development relative to root growth, which ultimately resulted in greater leaf biomass production under the supplementation of the biostimulant (Si-bioB).

#### 3.1.2. Shoot Fresh and Dry Weight

Results indicate that shoot fresh weight is significantly increased at the minimum application rates of Si-bioB (0.01X and 0.1X)—by 11% when compared to controls. Likewise, shoot dry weight also increased at low application rates (0.01X and 0.1X) of Si-bioB by 11–12% compared to untreated plants (Table 2).

In contrast, basil shoot fresh weight using Si-bioA showed a non-significant increase at different application rates (0.01X, 0.01X, 1X, and 2X) of 4–9% compared to controls, while Si treatments using Si-bioA at high application rates of 1X and 2X showed an increment in shoot dry weight (g) by 10–12% as compared to untreated controls (Table 2).

#### 3.1.3. Number of Leaves

The number of basil leaves per pot using Si-bioA (0.01X, 0.01X, 1X, and 2X) was not significantly different from each other and from untreated controls. However, a significant reduction of basil leaves by 45% was recorded using Si-bioB at the lowest application rate (0.01X) when compared to other application rates of Si-bioB, as well as the controls. In contrast, a significant increase was noted in the number of leaves at a high application rate of Si-bioB (2X)—by 19% compared to control plants (Table 2). Basil leaves under this treatment (T9) exhibited morphological differences, discoloration, a mottled appearance, and reduced leaf size, which may indicate crop stress at 2X (double the commercial application rate).

### 3.2. Physiological Parameters

#### 3.2.1. Relative Water Content (RWC)

**Figure 3:**
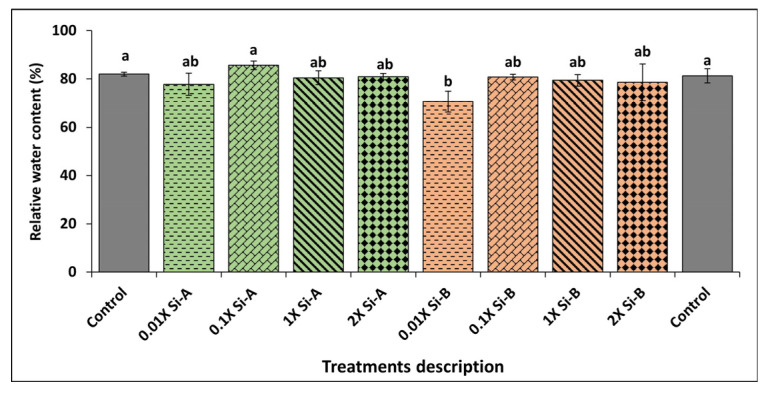
Effect of Si supplementation on the relative water content of basil growing under glasshouse conditions as affected by two Si-based biostimulants (Si-bio) applied at 4 application rates compared to untreated controls. Different letters indicate statistical significance (*p* < 0.05) as determined by the LSD test. Treatments: Si-A (SiO_2_, chelated Fe); Si-B (silicic acid tetraethyl ester); control: untreated plants.

RWC remained relatively consistent across controls and treatments except for the 0.01X Si-bioB, which showed a significant reduction in RWC (16%) compared to controls (Figure 3).

#### 3.2.2. Membrane Stability Index (MSI) and Electrolyte Leakage (EL)

**Figure 4:**
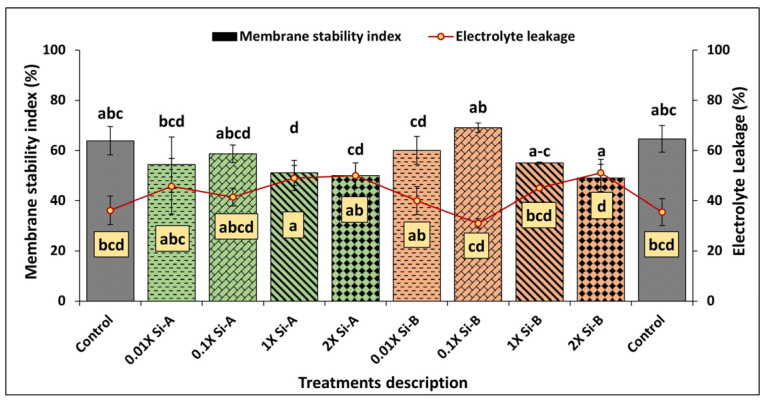
Effect of Si supplementation on the membrane stability index (MSI) and electrolyte leakage (EL) of basil growing under glasshouse conditions as affected by two Si-based biostimulants (Si-bio) applied at 4 application rates compared to untreated controls. Different letters indicate statistical significance (*p* < 0.05) as determined by the LSD test. Treatments: Si-A (SiO_2_, chelated Fe); Si-B (silicic acid tetraethyl ester); control: untreated plants.

For MSI and EL, basil receiving Si-bioB (silicic acid tetraethyl ester) treatments performed better overall than treatments of Si-bioA (SiO_2_ and chelated Fe). Treatment with a double commercial application rate (2X) of Si-bioB resulted in a significant enhancement of MSI by 9% and a reduction in electrolyte leakage by 22% (Figure 4). However, a significant decrease was observed in the MSI at the standard commercial and double commercial rates of Si-bioA (i.e., 1X and 2X), by 36% and 28%, respectively, with a corresponding increase in electrolyte leakage of 32% and 28%, respectively, compared with control plants. In contrast, at low application rates of Si-bioA (0.01X and 0.1X), MSI was reduced by 18% and 10%, and EL increased by 22% and 14% compared with untreated controls (Figure 4).

### 3.3. Photosynthetic Parameters

#### 3.3.1. SPAD Index

**Figure 5:**
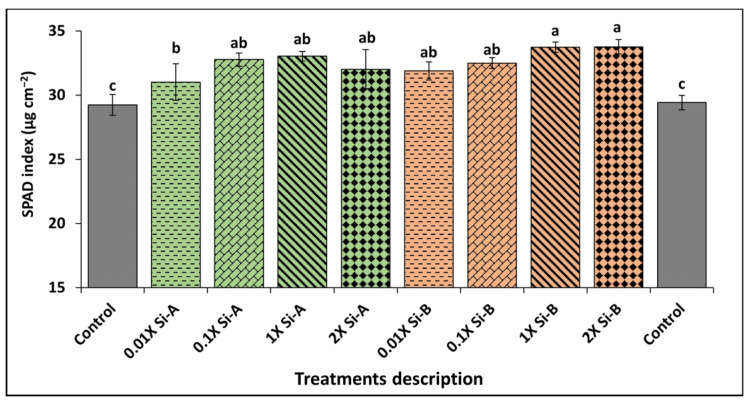
Effect of Si supplementation on the SPAD index of basil growing under glasshouse conditions as affected by two Si-based biostimulants (Si-bio) applied at 4 application rates compared to untreated controls. Different letters indicate statistical significance (*p* < 0.05) as determined by the LSD test. Treatments: Si-A (SiO_2_, chelated Fe); Si-B (silicic acid tetraethyl ester); control: untreated plants.

Consistently, there was a significant increase in relative photosynthetic activity as indicated in the SPAD index results with biostimulant supplementation using both Si-based products. Specifically, Si-bioB treatments at four application rates (0.01X, 0.1X, 1X, and 2X) enhanced the SPAD index by 7–12% compared to control plants. Likewise, supplementation with Si-bioA at 0.01X, 0.1X, 1X, and 2X also showed significant improvements in the SPAD index of 4%, 9%, 10%, and 7%, respectively, compared with untreated controls (T1 and T10) (Figure 5).

#### 3.3.2. Total Chlorophyll Content and Carotenoids

**Figure 6:**
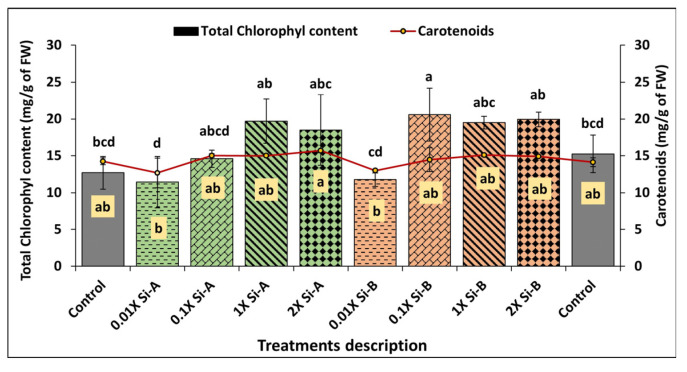
Effect of Si supplementation on the total chlorophyll content and carotenoids of basil growing under glasshouse conditions as affected by two Si-based biostimulants (Si-bio) applied at 4 application rates compared to untreated controls. Different letters indicate statistical significance (*p* < 0.05) as determined by the LSD test. Treatments: Si-A (SiO_2_, chelated Fe); Si-B (silicic acid tetraethyl ester); control: untreated plants.

Similarly, total chlorophyll content showed significant improvement with biostimulants using both Si-bio; Si-bioA at 1X and 2X by 29% and 24%, respectively, and Si-bioB at 0.1X, 1X, and 2X showed significant increases in total chlorophyll content by 32%, 28% and 30%, respectively, when compared to untreated plants in control treatments (T1 and T10). However, no differences in carotenoids were detected; they remained statistically similar to those of controls, indicating no effect of biostimulant supplementation on this parameter (Figure 6).

## 4. Discussion

The use of Si as a biostimulant has attracted increasing attention due to its potential to enhance plant growth, productivity, and stress resilience. Research on rice, wheat, and spinach has shown that silicon biostimulant supplementation can enhance crop growth under outdoor soil conditions. The current study contributes to this knowledge base by presenting a comprehensive assessment of the effects of a silicon biostimulant on sweet basil productivity and growth parameters, including agronomic, physiology, and photosynthetic efficiency, under glasshouse conditions. These findings provide valuable insights into the effects of varying silicon supplementation rates and propose an optimal application rate for enhancing basil cultivation.

Silicon products with different chemical forms exhibit distinctly different dose–response patterns. The two silicon products differ in their formulation. Si-BioA is a silicon form combined with chelated iron (Fe) providing both silicon and Fe to the plant. In contrast, Si-BioB is a form of organic soluble silicon. Notably, the RL:SL in Si-BioA treatments were greater across all dosages than Si-BioB. This suggests basil plants dedicated more resources to root growth which may signify that iron supply triggers lateral root elongation in a similar way to research findings by Giehl et al. [43], explained as a stress response to high levels of Fe.

The size and number of leaves were also improved with Si-bioA (increased at all application rates), showing positive effects on the morphology of basil leaves. These findings are consistent with research on leafy vegetables such as Ssamchoo, in which Si-enriched nutrient solutions increased leaf area and number [44]. Interestingly, Si supplementation with Si-bioB (at 2X: double commercial application rate) appeared to be excessive, resulting in smaller and mottled leaves. This response may be part of the plant’s adaptive mechanism to environmental stressors, in which, in some cases, plants increase leaf production under stress to enhance photosynthetic capacity and ensure survival [45]. Alternatively, the high levels of silicates in this biostimulant formulation may trigger a pH increase, limiting nutrient uptake and stunting growth [46].

Moreover, Si treatment improved basil yield parameters, including fresh and dry weight, with the most pronounced effects observed at the lowest Si-bioB application rate (0.01X). These results align with previous studies demonstrating Si’s role in promoting agronomic performance. For instance, Laîné et al. [47] report that Si enhances yield and nutrient use efficiency in Brassica crops, while Galindo et al. [48] show similar benefits in maize and wheat, where Si supplementation supported healthy growth and increased productivity of crops. Additionally, ALKahtani et al. [49] report that Si supplementation significantly increased both the yield and quality of lettuce, indicating that Si can mitigate stress effects and improve physiological traits, thereby conferring salt tolerance in leafy greens. These findings collectively reinforce the potential of Si as a sustainable tool for enhancing crop performance across diverse food crops.

Si supplementation is often application-rate dependent, with both insufficient and excessive application rates leading to suboptimal physiological responses [50]. The small sample sizes investigated for physiological parameters in this study limit interpretation to data trends rather than strong evidence. The results demonstrate an interesting trend: a non-significant reduction in RWC at the lowest Si application (0.01X) using Si-bioB. Compared to other treatments where RWC was good enough, our findings suggest that Si supplementation may be effective in managing the water deficit of the leaf [39], and where application rates are too low (e.g., 0.01X Si-bioB), the plant experiences an inability for osmotic adjustment and enhanced water retention under stress [51].

Our results further suggest that Si improves the membrane stability index (MSI) and lowers electrolyte leakage (EL) in basil leaves. Another study demonstrates that Si application can enhance the immune response of basil, suggesting potential improvements in MSI, particularly under stress conditions [30]. Robatjazi et al. [31] report that Si supplementation improved membrane integrity in basil plants subjected to salt stress. Our findings offer support to the idea that Si treatment can enhance MSI in basil plants, even under controlled conditions without stress, by improving membrane integrity and overall plant resilience. This enhancement in MSI is an essential parameter for assessing product shelf life during the post-harvest period of food [52,53].

Our results further indicate that Si biostimulant supplementation at all rates significantly enhances photosynthetic attributes, as reflected by higher SPAD index and total chlorophyll content, while leaving carotenoids unchanged relative to controls. Another study of various wheat cultivars found that Si treatments increased the SPAD index and enhanced growth under drought- and heat-stress conditions [54]. Similar to the SPAD index, analysis of total chlorophyll content also showed a significant increase under Si supplementation compared with control plants, thereby enhancing photosynthetic efficiency and overall basil crop health. Zhang et al. [55] demonstrate in a study on tomatoes that Si application increased chlorophyll and carotenoid content, thereby improving plant growth and stress tolerance under high-temperature and water-deficit conditions. Likewise, Li et al. [56] documented that Si supplementation significantly increased total chlorophyll content and carotenoids in Chinese cabbage. In this research, it is proposed that increasing the level of photosynthetic pigments optimize photosynthetic performance, which is linked to improved plant growth and development under both normal and stress conditions.

Table 3 summarizes plant responses to Si-based biostimulants, indicating that lower application rates of Si-bioB (0.01X and 0.1X) significantly improved basil growth and development.

The study shows the seemingly contradictory phenomenon of low doses promoting growth but potentially worsening some physiological indicators, while high doses inhibit growth but improve stress resistance indicators. This divergence is best described as a trade-off between growth and defense (“growth-defense trade-off”) which has a well-established basis in plant science literature [57]. This is explained as a molecular adjustment in plants linked to hormone crosstalk (i.e., signaling pathways in plants). This allows plants to adjust growth and defense based on external conditions including excessive silicon or Fe uptake [58].

## 5. Conclusions

The current findings present the effects of Si-based biostimulant supplementation on basil seedling growth and a single harvest. Despite the small sample sizes evaluated in certain parameters e.g., physiological parameters, the data overall provides evidence that Si supplementation in sweet basil production leads to a positive effect on traits linked to agronomic performance (morpho-physiological traits) and photosynthetic ability in particular. Our paper provides new data on the efficacy of two Si-based biostimulant products with different formulations. In doing so, three contributions to new knowledge are made:

(1) Under glasshouse production of sweet basil, the Si-based biostimulant silicic acid tetraethyl ester (0.01X and 0.1X dosages) can be used to promote growth.
(2) The commercial recommended dose (1X) was not optimal for multiple indicators in this study, highlighting the potential risks of high doses/wasteful application.
(3) The efficacy of Si-based biostimulants is highly dependent on the products’ chemical form and application dose. Targeted dose optimization trials are essential prior to application.

## Figures and Tables

**Table 1:** A summary of the treatment labels, silicon biostimulant, and application rates used in the pot experiment.

Treatments *		Application Rate	Si Concentration
T1	Untreated control	Without Si treatment	0.000%
T2	Si-bioA 0.01X	10 mL/ha	0.001%
T3	Si-bioA 0.1X	100 mL/ha	0.010%
T4	Si-bioA 1X	1 L/ha	0.100%
T5	Si-bioA 2X	2 L/ha	0.200%
T6	Si-bioB 0.01X	10 mL/ha	0.0017%
T7	Si-bioB 0.1X	100 mL/ha	0.017%
T8	Si-bioB 1X	1 L/ha	0.170%
T9	Si-bioB 2X	2 L/ha	0.340%
T10	Untreated control	Without Si treatment	0.000%

Treatments *: Si-bioA: SiO_2_ and chelated Fe; Si-bioB: silicic acid tetraethyl ester; untreated control: plants without Si application.

**Table 2:** Effect of Si supplementation on the agronomic attributes of basil growing in peat under glasshouse conditions as affected by two Si-based biostimulants (Si-bio) applied at 4 application rates and non-treatment controls.

Treatments *		SL (cm)	RL (cm)	SFW (g)	SDW (g)	NOL	RL:SL
**T1**	Untreated control	18.17 ± 0.96 **^bc^**	13.25 ± 1.44 **^c^**	26.67 ± 0.33 **^b^**	2.16 ± 0.06 **^c^**	50 ± 3.19 **^cd^**	0.72
**T2**	Si-bioA 0.01X	19.67 ± 0.76 **^ab^**	15.32 ± 1.25 **^ab^**	27.67 ± 1.61 **^ab^**	2.16 ± 0.17 **^c^**	45 ± 3.37 **^d^**	0.78
**T3**	Si-bioA 0.1X	19.67 ± 0.83 **^ab^**	15.83 ± 1.05 **^ab^**	28.50 ± 1.02 **^ab^**	2.18 ± 0.09 **^bc^**	49 ± 1.83 **^cd^**	0.81
**T4**	Si-bioA 1X	18.58 ± 0.49 **^abc^**	17.42 ± 0.85 **^a^**	29.17 ± 0.70 **^ab^**	2.45 ± 0.06 **^ab^**	50 ± 2.23 **^cd^**	0.94
**T5**	Si-bioA 2X	16.58 ± 0.64 **^c^**	14.00 ± 1.15 **^bc^**	28.33 ± 0.99 **^ab^**	2.49 ± 0.10 **^a^**	50 ± 2.36 **^cd^**	0.84
**T6**	Si-bioB 0.01X	20.25 ± 0.89 **^a^**	15.17 ± 0.94 **^ab^**	29.67 ± 1.41 **^a^**	2.47 ± 0.13 **^ab^**	36 ± 2.26 **^e^**	0.75
**T7**	Si-bioB 0.1X	19.75 ± 1.15 **^ab^**	12.67 ± 0.65 **^c^**	29.67 ± 0.95 **^a^**	2.51 ± 0.05 **^a^**	46 ± 3.36 **^d^**	0.64
**T8**	Si-bioB 1X	18.25 ± 0.44 **^abc^**	13.17 ± 0.53 **^c^**	27.50 ± 0.99 **^ab^**	2.29 ± 0.08 **^abc^**	58 ± 4.30 **^ab^**	0.72
**T9**	Si-bioB 2X	18.58 ± 0.37 **^abc^**	13.25 ± 0.59 **^bc^**	26.33 ± 1.12 **^b^**	2.09 ± 0.14 **^c^**	65 ± 1.92 **^a^**	0.74
**T10**	Untreated control	17.75 ± 0.89 **^bc^**	13.17 ± 1.38 **^bc^**	26.33 ± 0.80 **^b^**	2.25 ± 0.09 **^abc^**	56 ± 2.41 **^bc^**	0.78

Values represent the mean of each treatment for an agronomic parameter with different superscript bold letters representing statistical significance of *p* < 0.05 as determined by the LSD test and ± indicates standard errors of the mean of three replicates. Treatments *: Si-bioA: SiO_2_, chelated Fe; Si-bioB: silicic acid tetraethyl ester; untreated control: plants grown without Si application. Shoot length = SL; root length = RL; number of leaves = NOL; shoot fresh weight = SFW; and shoot dry weight = SDW.

**Table 3:** Summary of parameters showing a significantly improved response to silicon-based biostimulant products.

Si-Based Biostimulant Application Rate		Si-BioA				Si-BioB			
		0.01X	0.1X	1X	2X	0.01X	0.1X	1X	2X
Parameters	Agronomic	√√	√√	√√	√	√√√√√	√√√	−	−
	Physiology	−	−	−	−	−	√√	−	√√
	Photosynthesis	√	√√	√√	√√	√	√√	√√	√√
	Overall Performance	−	*	*	−	**	***	−	*

Signs indicated “√” presence and “−“absence of parameter/s under the effect of two Si-based biostimulants. Improvement level/s: * low level; ** medium level; *** high level. Si-bioA: SiO_2_, chelated Fe; Si-bioB: silicic acid tetraethyl ester.

## Data Availability

The data presented in this study are available on request from the corresponding authors due to to institutional and project data-sharing restrictions.

## Supplementary Materials

The following supporting information can be downloaded at: https://www.mdpi.com/article/10.3390/plants15060859/s1.
